# 3D Culture Reverses Limbal Niche Cell Replicative Aging via FOSL1 Upregulation

**DOI:** 10.1111/acel.70705

**Published:** 2026-09-08

**Authors:** Xuying Wang, Shen Li, Zibin Liu, Xinghan Guo, Jiachao Shen, Tianyu Zhou, Shuying Liao, Xiaoyu Huang, Wei Wang, Lingjuan Xu, Xinjie Zang, Guigang Li

**Affiliations:** ^1^ Department of Ophthalmology, Tongji Hospital, Tongji Medical College Huazhong University of Science and Technology Wuhan Hubei China; ^2^ Hubei Key Laboratory of Otorhinolaryngologic and Ophthalmic Diseases Wuhan Hubei China; ^3^ Department of Ophthalmology University Hospital of Erlangen, Friedrich‐Alexander‐University of Erlangen–Nuremberg (FAU) Erlangen Germany; ^4^ Chiemsee Augen Tagesklinik Prien Germany; ^5^ Department of Ophthalmology Qilu Hospital of Shandong University (Qingdao) Qingdao Shandong China

**Keywords:** 3D cell culture, cellular senescence, FOSL1, limbus, mitochondrial homeostasis

## Abstract

Limbal niche cells (LNCs) serve as essential regulators of limbal microenvironmental homeostasis and corneal epithelial wound repair, representing a promising therapeutic resource for limbal stem cell deficiency (LSCD). However, their clinical application is constrained by replicative aging during in vitro expansion. In this study, we investigated whether a three‐dimensional (3D) Matrigel‐based culture system could modulate replicative aging in LNCs. Compared with conventional two‐dimensional (2D) culture, 3D‐cultured LNCs restored stemness marker expression and enhanced proliferative capacity. Concurrently, these cells displayed reduced senescence‐associated β‐galactosidase (SA‐β‐gal) activity and decreased expression of senescence‐associated proteins, including p16, p21, p53, and γ‐H2AX. Single‐cell RNA sequencing (scRNA‐seq) analysis revealed prominent upregulation of FOS‐like antigen 1 (FOSL1). FOSL1 is a component of the AP‐1 transcription factor family and participates in cell proliferation and stress adaptation. Functional assays using an in vitro replicative aging model showed that FOSL1 knockdown in early‐passage (P4) LNCs accelerated senescence, whereas FOSL1 overexpression in late‐passage (P11) LNCs attenuated senescence. Mechanistically, FOSL1 knockdown induced mitochondrial dysfunction characterized by elevated levels of mitochondrial superoxide and cellular reactive oxygen species (ROS), as well as a decrease in mitochondrial membrane potential, while FOSL1 overexpression preserved mitochondrial integrity and function. Collectively, our findings demonstrate that 3D culture reverses LNC replicative aging through FOSL1‐mediated enhancement of mitochondrial function, providing a microenvironment‐based strategy to counteract replicative aging in adult stem cells for corneal regenerative therapy.

## Graphical abstract

3D culture reverses LNC replicative aging via FOSL1 upregulation, enhancing mitochondrial function and alleviating oxidative stress.

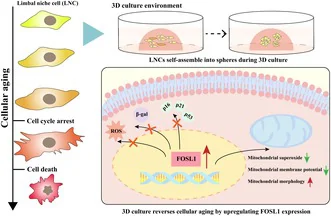

## 1 Introduction

The transparency of the cornea and good visual function depend on a healthy reservoir of limbal epithelial stem cells (LESCs), a cell population also known as limbal stem cells (LSCs) (Bonnet et al. 2021). Deficiency or dysfunction of LESCs disrupts the regeneration of the corneal epithelium, leading to limbal stem cell deficiency (LSCD). LSCD is a refractory ocular surface disease characterized by conjunctivalization of the corneal epithelium, which prevents the epithelial wound from healing and severely impairs vision (Deng et al. 2019). LESCs are situated at the limbus, clinically recognized as the palisades of Vogt. LESCs are regulated by multiple factors in this microenvironment, including non‐epithelial cell components, extracellular matrix (ECM) components, blood vessels, and nerves (Li et al. 2007; Ordonez and Di Girolamo 2012). Among these microenvironmental components, limbal niche cells (LNCs) serve as critical regulators. LNCs are derived from the anterior stroma adjacent to the limbal epithelium and exhibit mesenchymal stem cell (MSC) characteristics (Li et al. 2012; Xie et al. 2012; Li, Sun, et al. 2025). In vitro co‐culture studies demonstrated that LNCs induced the dedifferentiation of mature corneal epithelial cells by downregulating CK12 and upregulating p63α (Zhu et al. 2022). In a rabbit model of alkali burn‐induced LSCD, subconjunctival injection of LNCs exhibited superior therapeutic effects relative to bone marrow mesenchymal stem cells (BMSCs), reducing epithelial defects, corneal neovascularization, and goblet cell infiltration (Li et al. 2018). Additionally, LNCs promoted scarless corneal healing by inhibiting neutrophil migration and preventing fibrotic tissue deposition (Hertsenberg et al. 2017; Basu et al. 2014). LNCs hold great potential for clinical applications in the treatment of LSCD and other corneal diseases.

Currently, cellular senescence is defined as an irreversible arrest of cell proliferation. This state is accompanied by profound changes in cellular morphology, secretory phenotype, and the epigenetic landscape (van Deursen 2014; Gorgoulis et al. 2019). However, cellular changes occur progressively and continuously long before complete cell cycle arrest. In this study, we use cellular aging to describe a broader process of progressive functional decline (Ogrodnik 2021). Replicative aging refers to cellular aging that occurs during in vitro expansion. To acquire adequate cell quantities for clinical applications, cells must be expanded through serial passaging. However, replicative aging limits the expansion of LNCs (Hayflick and Moorhead 1961). This process is characterized by increased intracellular expression of p21, p16, and p53, and enhanced SA‐β‐gal activity (Ogrodnik 2021). Mitochondria are the centers of cellular energy metabolism and play a central role in cellular aging (Wang, Yuan, et al. 2024; Passos et al. 2007). Mitochondrial function gradually declines as cells continue to divide, manifesting as impaired mitochondrial respiration, reduced adenosine triphosphate (ATP) production, and metabolic dysfunction. Additionally, reactive oxygen species (ROS) are generated as byproducts during mitochondrial energy production (Lesnefsky and Hoppel 2006). Excess ROS damages mitochondrial DNA (mtDNA), causing damage to genetic material (Bratic and Larsson 2013; López‐Otín et al. 2013). At the same time, mitochondrial proteins undergo oxidative modification. The efficiency of the electron transport chain consequently declines, and the quality control mediated by mitochondrial autophagy becomes difficult to maintain. These lesions accumulate continuously, gradually leading to widespread changes in gene expression profiles, metabolic activity, and cell morphology. Ultimately, cell proliferation comes to a halt. Hence, reversing replicative aging in LNCs is essential for clinical applications.

For decades, two‐dimensional (2D) culture has remained the mainstream method for in vitro cell expansion. Traditional 2D systems struggle to replicate the true in vivo microenvironment. When stem cells are cultured on flat surfaces for extended periods, they often gradually lose their original characteristics and develop functional impairments (Jiang et al. 2017). Consequently, researchers have continuously explored new alternatives. Among numerous attempts, three‐dimensional (3D) culture has demonstrated promising applications (Badr‐Eldin et al. 2022). Under 3D conditions, MSCs form spheroids and exhibit enhanced adaptive capacity, augmented secretory profiles, and potentiated immunomodulatory functions (Murphy et al. 2017; Bartosh et al. 2010). Notably, 3D culture has been shown to ameliorate replicative aging phenotypes in stem cells (Chen et al. 2020; Yin et al. 2020; Shi et al. 2024). Our previous study showed that when LNCs propagated to the fourth generation were reseeded onto a Matrigel 3D scaffold, the expression of their embryonic stem cell markers significantly rebounded (Xie et al. 2012). Building upon these findings, we hypothesized that 3D culture would confer protective effects against replicative aging of LNCs.

FOS‐like antigen 1 (FOSL1) is an AP‐1 family transcription factor. It participates in cell proliferation, differentiation, and stress adaptation (Maurus et al. 2017). Recent studies showed that FOSL1 promotes stem cell properties by regulating SOX2 transcription (Wang, Hu, et al. 2024). In the corneal epithelium, FOSL1 is robustly expressed in LESCs and maintains their stemness (Adhikary et al. 2005). It remains to be determined whether FOSL1 is involved in cellular aging.

We observed that LNCs could self‐assemble into spheroids in 3D culture. Compared with traditional 2D monolayer culture, 3D‐cultured LNCs exhibited restored expression of stem cell markers, enhanced proliferative capacity, and reduced expression of aging‐related markers. To further characterize these changes, single‐cell RNA sequencing (scRNA‐seq) was performed, and differential gene expression analysis identified FOSL1 as a potential key signaling regulator associated with replicative aging. We then manipulated FOSL1 expression in an in vitro replicative aging model and demonstrated that FOSL1 overexpression attenuated replicative aging, whereas FOSL1 knockdown had the opposite effect. Furthermore, our results revealed that FOSL1 enhanced mitochondrial function. These findings provide novel understanding into the effects of 3D culture on replicative aging and enhance the clinical translational potential of LNCs.

## 2 Methods

### 2.1 LNC Isolation and Culture

Corneal tissues were obtained from the Red Cross Eye Bank at Tongji Hospital in Wuhan, China, from donors aged 60–80 years. The use of specimens adhered to the Declaration of Helsinki. The study received ethical approval from the Institutional Review Board at Tongji Hospital (TJ‐IRB20230899). Detailed donor information is provided in Table S1.

The method for isolating and culturing LNCs was consistent with that used in our previous studies (Xie et al. 2012; Zhou et al. 2025). Excised limbal tissues were digested using 1 mg/mL collagenase A (Roche, Basel, Switzerland) in 3.5 cm dishes for 16–18 h to harvest epithelial–stromal cell clusters. After enzymatic dissociation with 0.25% trypsin/0.5 mM EDTA at 37°C for 10 min, cells were collected and plated on six‐well dishes and expanded in modified embryonic stem cell medium (MESCM) in the incubator at 37°C with 5% CO_2_. MESCM comprised DMEM supplemented with 10% Knockout Serum Replacement, 50 μg/mL basic fibroblast growth factor, 25 μg/mL gentamicin, 100 μg/mL leukemia inhibitory factor, 500 μg/mL ITS (Insulin‐Transferrin‐Selenium), and 250 μg/mL amphotericin B. LNCs were split and reseeded at 5 × 10^3^ cells/cm^2^ once they reached 80%–90% confluence.

### 2.2 Matrigel Pre‐Coating

Cell culture plates were coated with 5% Matrigel prior to use. Matrigel was thawed at 4°C. Pre‐chilled 200‐μL and 1000‐μL pipette tips, 15 mL centrifuge tubes, and cell culture plates were placed at −80°C for at least 20 min. Pre‐chilled MESCM (500 μL) and Matrigel (25 μL) were added to a centrifuge tube and mixed thoroughly. The mixture was added to the culture plate. The plate was gently shaken to ensure the gel covered the bottom of the wells and then put in the incubator, after which LNCs were seeded.

### 2.3 Construction of 3D Cultivation System

A 24‐well plate was pre‐warmed in an incubator for 30 min prior to cell seeding. The LNC suspension was mixed with Matrigel (Corning, USA) at a 1:1 volume ratio to prepare a solution containing 50% Matrigel and 8 × 10^4^ cells/mL. Then 50 μL of this mixture was slowly dispensed into the center of each well to form a hemispherical droplet. The culture plate was inverted and incubated for 10 min to promote gelation and prevent cell sedimentation. Subsequently, 1 mL of MESCM was carefully added along the edge of each well to avoid mechanical disturbance. A 50% medium change was performed every 72 h. The morphology and growth of the spheroids were recorded daily, and the cells were harvested on Day 6 for subsequent experiments.

### 2.4 Paraffin Embedding and Slicing

Corneal tissues were immersed in 4% paraformaldehyde for 48 h. The specimens were rinsed with PBS for 30 min and processed sequentially through a gradient of ethanol, xylene, and paraffin. After paraffin infiltration and embedding, 4‐μm‐thick sections were cut and placed onto slides. These slides were dried at 60°C for 2 h. The prepared sections were stored for later use.

### 2.5 Immunofluorescence (IF) Staining

- *2D cultured LNCs*. For cytospins, cells were digested and centrifuged, then resuspended in PBS. They were centrifuged at 1000 rpm for 4 min using a StatSpin CytoFuge 2 (Beckman Coulter, Brea, CA, USA) and spread onto slides (1 × 10^4^ cells per slide). For coverslips, isolated cells were suspended in MESCM and seeded onto sterile coverslips in a 24‐well plate (1 × 10^4^ cells per well). The plates were cultivated for 24 h. Then the coverslips were removed for staining.
- *3D spheroids*. Dispase II was added to culture plates and incubated at 37°C for 10 min to digest the Matrigel. The suspension was centrifuged at 1000 rpm for 5 min, and the collected pellet was incubated with 0.25% trypsin–EDTA at 37°C for 5 min. MESCM was added to stop digestion. Finally, the cells were spun down onto slides or plated onto coverslips in 24‐well plates at a density of 1 × 10^4^ cells per well, and maintained for 24 h.
- *Cornea paraffin sections*. The paraffin on the surface of the tissue sections was melted. The sections were then deparaffinized using xylene and a gradient of ethanol. Heat‐induced epitope retrieval (HIER) was conducted in EDTA buffer (pH 9.0).

#### 2.5.1 Immunostaining Procedure

For intracellular antigens, the cell membrane was permeabilized with 0.01% Triton X‐100 at room temperature for 15 min. All samples were blocked with 10% donkey serum for 1 h. Subsequently, the primary antibody was diluted to the recommended concentration using the blocking serum and incubated overnight at 4°C. Three TBST washes were performed. Then the samples were incubated with fluorescently labeled secondary antibody at 37°C for 45 min. DAPI (4′,6‐diamidino‐2‐phenylindole; Solarbio, China) was used as a nuclear counterstain. Images were taken under a fluorescence microscope (LSM 700; Carl Zeiss Microscopy, Jena, Germany) and processed using NIS‐Elements software (Nikon, Tokyo, Japan). Comprehensive antibody data are provided in Table S2.

### 2.6 Western Blot (WB) Analysis

RIPA buffer (Beyotime, China) containing 1% PMSF and protease inhibitors was added to the plates. LNCs were lysed on ice for 30 min. The lysate was centrifuged at 12,000 *g* for 10 min at 4°C to pellet cellular debris. The protein concentration in the clear supernatant was determined using the BCA protein assay (Beyotime, China). For electrophoresis, the protein extract was mixed with 5× loading buffer at a 4:1 volume ratio and heat‐denatured at 95°C for 5 min. Separation was performed on a 10% SDS‐PAGE gel (Biossci, China) at a constant voltage of 100 V. The proteins were transferred to a PVDF membrane (Millipore, USA; 300 mA, 90 min), blocked in 5% skim milk (1 h), probed with primary antibody (4°C, overnight), and rinsed with TBST to remove unbound antibody. The HRP‐labeled secondary antibody was diluted 1:5000 in TBST and incubated with the membrane for 1 h, followed by three washes. Signals were detected using an ECL chemiluminescent detection kit. Detailed antibody information is provided in Table S2.

### 2.7 Cell Proliferation Assay

The proliferative activity of LNCs was determined using the CCK‐8 assay (Shanghai University, China). Cells were dissociated and seeded at a density of 5 × 10^3^ cells per well into 96‐well plates pretreated with Matrigel (5%). At 4, 24, 48, and 72 h post‐seeding, 10 μL of CCK‐8 solution was added. Absorbance was measured at 450 nm using a microplate reader. All readings were normalized against the 4 h control group to calculate the relative proliferation rate.

### 2.8 SA‐β‐Gal Staining Assay

SA‐β‐gal staining (Beyotime, Shanghai, China) was used to identify senescent cells. For adherent cells, staining is performed directly in the culture plate. For 3D‐cultured cells, the cells are first processed into single cells, seeded in a culture plate, and cultured for 24 h before staining. After discarding the culture supernatant, the cells were fixed for 15 min using SA‐β‐gal fixative. After fixation, the cells underwent three 3‐min washes with PBS. A fresh staining mixture was prepared by combining 10 μL of Solution A, 10 μL of Solution B, 930 μL of Solution C, and 50 μL of X‐Gal per well. The culture plates were incubated at 37°C in a CO_2_‐free environment for 24 h and sealed with Parafilm to prevent solution evaporation. After removing the staining solution and rinsing with PBS, blue‐stained senescent cells were counted using a bright‐field microscope.

### 2.9 RT‐qPCR Analysis

RNA extraction was performed with Trizol (Sigma, USA) following the standard chloroform‐isopropanol protocol. Briefly, cells were homogenized in 1 mL Trizol, mixed with 200 μL chloroform (Sinopharm, China), and the aqueous layer was precipitated with isopropanol (Sinopharm, China). After ethanol washing (Biossci, Wuhan, China), RNA was resuspended in nuclease‐free water (55°C, 5 min). Quality was verified by spectrophotometry (Rayto, Shenzhen, China). Reverse transcription was conducted using a Vazyme kit: gDNA removal at 42°C for 2 min, cDNA synthesis at 50°C for 15 min, and inactivation at 85°C for 2 min. qPCR (20 μL) included an initial denaturation at 95°C for 1 min, 40 amplification cycles (95°C for 10 s, 60°C for 30 s), and a melting curve stage (60°C–95°C, ramp 0.3°C/15 s). Expression levels were normalized to GAPDH and quantified by the $2^{-∆∆C_{t}}$ method. Primer details are provided in Table S3.

### 2.10 Single‐Cell RNA Sequencing (scRNA‐Seq) Analysis

LNC samples were washed in ice‐cold RPMI 1640 and dissociated into single‐cell suspensions using the Multi Tissue Dissociation Kit 1 (Miltenyi) following the manufacturer's protocol. Erythrocytes were removed, and cell viability was assessed under fluorescence microscopy. Viable cells were resuspended in PBS containing 0.04% BSA and processed using the BD Rhapsody Single‐Cell Analysis System. Polyadenylated mRNA was captured on oligo (dT) beads, and subjected to on‐bead reverse transcription and cDNA amplification. Amplified products were purified and used for library preparation. Libraries were sequenced on an Illumina NovaSeq 6000 platform (150 bp, paired‐end reads). Raw sequencing data were processed using the BD Rhapsody pipeline to generate the digital gene expression matrices. Downstream analysis was performed in R using Seurat (v4.4.0) with standard quality control metrics (≥ 1000 UMIs, ≥ 500 genes, < 20% mitochondrial reads), followed by doublet removal via DoubletFinder. Batch effects were corrected with Harmony. Cell clustering (resolution = 0.4), dimensionality reduction (UMAP/t‐SNE), and marker gene identification were performed using standard workflows. Differential expression analysis was conducted using Presto (|log_2_FC| > 0.5, adjusted *p* < 0.05), followed by functional enrichment analysis of GO terms and KEGG pathways using KOBAS 2.0. Pseudotemporal trajectory reconstruction was performed using Monocle2 with DDRTree dimensionality reduction and BEAM for branch‐specific gene regulation analyses. FOSL1‐associated genes and enriched biological processes were extracted from the STRING database (https://string‐db.org) and subsequently mapped using Cytoscape software. Raw sequencing reads were deposited in the NCBI Gene Expression Omnibus (GEO) under accession number GSE343251.

### 2.11 Transfection of siRNA

LNCs were plated at 5 × 10^5^ cells per well in six‐well plates and cultured for 24 h before transfection. FOSL1‐specific siRNA (50 nM; OBio Technology, Shanghai, China) was delivered using Lipofectamine 2000 (Invitrogen) according to the manufacturer's protocol. Briefly, siRNA and Lipofectamine 2000 (5 μL/well) were each diluted in 125 μL Opti‐MEM, combined, and allowed to complex for 20 min. The resulting mixture was then added to the cells. After 5 h, the medium was exchanged for fresh MESCM. A non‐targeting scrambled siRNA (OBio Technology) served as the negative control. Cells were collected at 48 h post‐transfection for qPCR and at 72 h for Western blot to confirm FOSL1 knockdown.

### 2.12 Plasmid Transfection

The complete coding sequence of human FOSL1 was inserted into the pcDNA3.1 (+) expression vector (OBio Technology, Shanghai, China). Transfection was performed on LNC cultures at 70%–90% confluence. Plasmid DNA (1–2 μg per well) was delivered using Lipofectamine 3000 reagent (Invitrogen) according to the supplier's recommended procedure. In brief, plasmid constructs were first diluted in 25 μL Opti‐MEM and complexed with P3000 enhancer reagent (1 μL per well). Concurrently, Lipofectamine 3000 transfection reagent was diluted in a separate 25 μL aliquot of Opti‐MEM at 1.5 μL per well. Following a 10–15 min equilibration period at ambient temperature, the DNA‐P3000 complex was merged with the Lipofectamine 3000 dilution. This transfection mixture was incubated for another 15 min at room temperature before being introduced in a dropwise manner onto the cell monolayer. The pcDNA3.1 (+) backbone without insert served as the mock transfection control. Functional characterization of transfected cells was performed 48 h after DNA delivery.

### 2.13 ROS Detection Assay

Cellular ROS content was examined with a commercial kit (ROS Assay Kit, Beyotime, S0033, Shanghai, China). This assay detects ROS through the oxidation of 2′,7′‐dichlorodihydrofluorescein diacetate (DCFH‐DA). In brief, LNCs were plated into 24‐well culture vessels and subjected to the designated experimental treatments. Prior to probe loading, cultures underwent two rinses with serum‐free medium to eliminate residual serum components. A 10 μM solution of DCFH‐DA prepared in DMEM was added, and the cells were incubated for 20 min at 37°C in the dark. Cells were then observed under a fluorescence microscope.

### 2.14 Mitochondrial Superoxide Detection Assay

MitoSOX Red (Beyotime, S0061S, Shanghai, China), a mitochondria‐targeted fluorescent probe, was used to assess mitochondrial superoxide production. Prior to staining, the cells were washed with PBS, followed by the addition of 5 μM MitoSOX Red prepared in DMEM. The cells were incubated for 30 min.

### 2.15 Mitochondrial Membrane Potential Assay

Mitochondrial membrane potential (ΔΨm) was assessed using the JC‐1 Assay Kit (Beyotime, C2005, Shanghai, China). LNCs were cultured in a 24‐well plate and rinsed with PBS, followed by the addition of 1 mL 1 × JC‐1 working solution in DMEM. Then the plates were maintained for 20 min at 37°C in the dark. Subsequently, the cells underwent three PBS washes to clear residual probe and were immediately observed under a fluorescence microscope.

### 2.16 Transmission Electron Microscopy (TEM)

Tissue processing began with overnight fixation in 4% glutaraldehyde at 4°C. After PBS washes, samples were stabilized in 2% osmium tetroxide (1 h, room temperature), dehydrated through graded ethanols, and embedded in Epon 812 (Serva). Sections of 70–80 nm were cut, mounted on copper grids, and double‐stained with uranyl acetate and lead citrate. Imaging was performed on a Tecnai G2 Spirit TEM (FEI) at 80 kV, with representative fields documented at 4000× and 10,000×.

### 2.17 Statistical Analysis

ImageJ and GraphPad Prism 8.0 were used for data analysis, and Adobe Illustrator for figure preparation. An independent *t*‐test was applied for two‐group comparisons. One‐way ANOVA was applied for multiple groups, and two‐way ANOVA for analyzing interactions between intervention groups and time points. Quantitative data are presented as mean ± standard deviation (SD) from three independent biological replicates. The significance thresholds were defined as follows: *p < 0.05; **p < 0.01; ***p < 0.001; ****p < 0.0001; ns = not significant.

## 3 Results

### 3.1 LNCs Self‐Assemble Into Spheroids During 3D Culture

**FIGURE 1:**
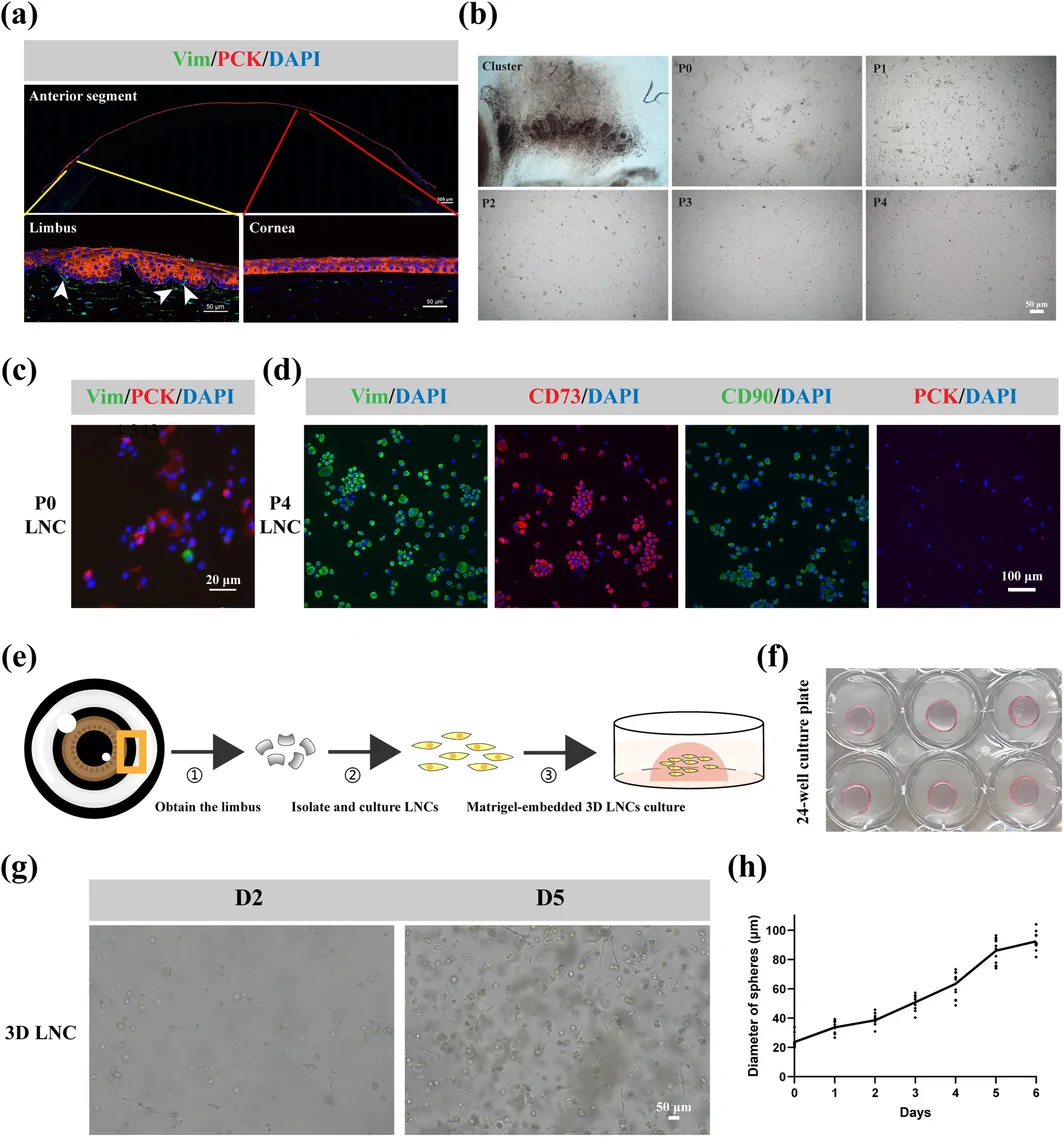
Isolation, purification, and 3D culture of LNCs. (a) IF staining of the anterior segment of the human cornea for PCK (red), Vim (green), and DAPI (blue). The images show magnified views of the cornea and the limbus, with the locations of LNCs indicated by white arrows. Scale bars, 500 μm (top image), 50 μm (bottom image) (*n* = 3). (b) Microscopic observation of digested limbal clusters and LNCs from P0 to P4. Scale bars, 50 μm (*n* = 3). (c) IF staining of P0 LNCs for PCK (red), Vim (green), and DAPI (blue) to evaluate the epithelial and stromal characteristics. Scale bar, 20 μm (*n* = 3). (d) IF staining of P4 LNCs for Vim (green), CD73 (red), CD90 (green), PCK (red), and DAPI (blue) to assess the purification efficiency. Scale bars, 50 μm (*n* = 3). (e) Schematic illustration of LNC isolation and 3D culture protocol. (f) Bright‐field image of hemispheric Matrigel droplets containing LNCs in a 24‐well plate at Day 0. (g) Representative images showing the morphology of LNC spheroids on days 2 and 5. Scale bars, 50 μm (*n* = 3). (h) LNC spheroid diameter during 6 days in 3D culture. *n* ≥ 3, biological replicates per group.

IF double staining of anterior segment tissues revealed the spatial relationship between epithelial and stromal cells (Figure 1a). In the corneal region, PCK‐positive cells constituted the corneal epithelium. In the limbal region, the epithelium was thickened, and the irregular basal lamina formed the palisades of Vogt. Vim‐positive cells were located within the limbal stroma, adjacent to the epithelial basement membrane. This indicated that the limbus possessed a unique structure with a more complex cellular composition. To obtain purified LNCs, limbal tissue clusters were isolated using enzymatic digestion and passaged to achieve purification. Observation under a phase‐contrast microscope revealed that, during the serial passage from P0 to P4, the adherent cells gradually transitioned from a heterogeneous, mixed state in P0 to a uniform spindle‐shaped morphology in P4 (Figure 1b). IF staining of P0 revealed that P0 contained both PCK‐positive epithelial cells and Vim‐positive stromal cells, representing a mixed pattern (Figure 1c). Following serial passaging to P4, cells showed negligible epithelial marker PCK expression but robustly expressed mesenchymal stem cell‐associated markers, including Vim, CD73, and CD90, indicating successful purification of LNCs (Figure 1d). To establish a 3D culture microenvironment, we developed a Matrigel‐based hemispheric droplet culture system (schematic illustration in Figure 1e). LNCs were cultured in suspension within 50% Matrigel droplets embedded in a 24‐well plate (Figure 1f). Under this 3D culture system, LNCs spontaneously formed spheroids. Microscopic observations on days 2 and 5 showed that the spheroids were gradually developing (Figure 1g). Quantitative analysis demonstrated that the spheroid diameter increased progressively over the 6‐day culture period, suggesting possible cellular proliferation and matrix remodeling within the 3D microenvironment (Figure 1h). These results confirmed that purified LNCs self‐assembled into 3D spheroids in a Matrigel‐based culture system.

### 3.2 3D Culture Reverses LNC Aging Compared With 2D Culture

**FIGURE 2:**
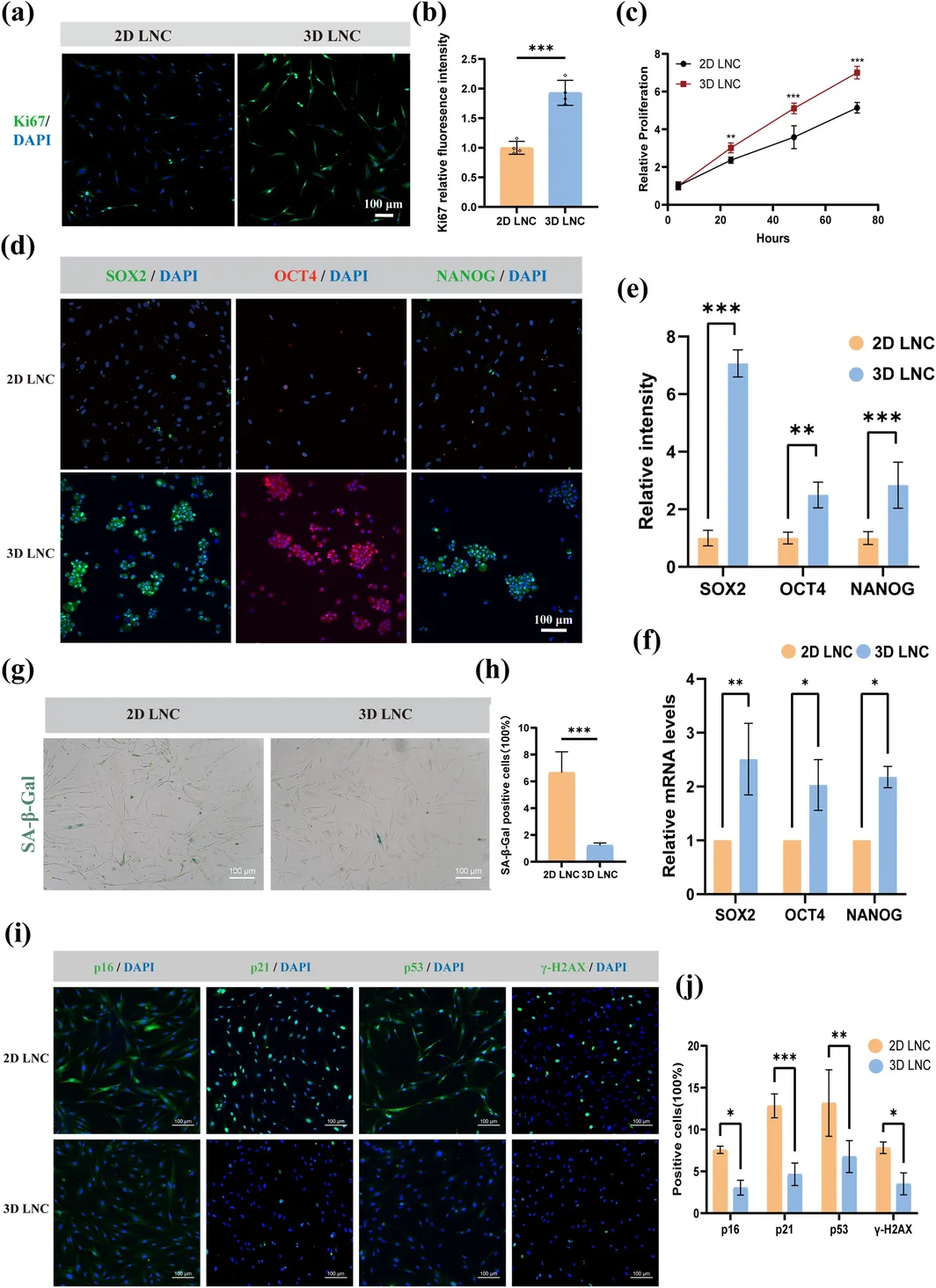
3D culture inhibits cellular aging. (a) IF images of Ki67 (green) and DAPI (blue) staining in 2D‐ and 3D‐cultured LNCs. Scale bars, 100 μm (*n* = 3). (b) Quantification of Ki67 relative fluorescence intensity. Data represent mean ± SD of *n* = 3. (c) Cell proliferation curve measured by CCK‐8 assay over 72 h (*n* = 3). (d) IF staining of stemness markers SOX2 (green), OCT4 (red), and NANOG (green) with DAPI (blue) counterstaining. Scale bars, 100 μm (*n* = 3). (e) Quantitative analysis of relative fluorescence intensity for SOX2, OCT4, and NANOG. Data represent mean ± SD of *n* = 3. (f) Relative mRNA expression levels of *SOX2*, *OCT4*, and *NANOG* were determined by RT‐qPCR. (g) Representative bright‐field images of SA‐β‐gal staining. Scale bars, 100 μm (*n* = 3). (h) Quantification of SA‐β‐gal‐positive cells (*n* = 3). (i) IF staining of senescence‐associated markers p16, p21, p53, and γ‐H2AX (all green) with DAPI (blue). Scale bars, 100 μm (*n* = 3). (j) Quantification of positive cells for p16, p21, p53, and γ‐H2AX, *n* = 3. Data are presented as mean ± SD from three independent experiments. Statistical significance was determined by Student's *t*‐test: ***p < 0.001; **p < 0.01; *p < 0.05.

To evaluate the effects of 3D culture on LNCs, we compared LNCs cultured under 2D condition with those cultured in a Matrigel‐based 3D system in terms of proliferation capacity, maintenance of stem cell properties, and cellular senescence. Cellular proliferation was measured by Ki67 IF and CCK‐8. 3D spheroids were dissociated into single cells, cultured on coverslips for 24 h, and then fixed and stained. Compared with 2D culture, the 3D‐cultured LNCs exhibited higher Ki67 relative fluorescence intensity, indicating enhanced proliferative activity (Figure 2a,b, *p* < 0.001). In the CCK‐8 assay, cells cultured under both conditions were seeded into a 96‐well plate at the same density, and absorbance was measured at 4, 24, 48, and 72 h. The results showed that, compared with the 2D control group, LNCs cultured in 3D exhibited stronger proliferative capacity (Figure 2c, *p* < 0.001). Then we performed IF staining to assess the expression of pluripotency markers SOX2, OCT4, and NANOG. IF staining results showed that, compared with 2D culture, the expression of SOX2, OCT4, and NANOG was significantly elevated in LNCs cultured in 3D (Figure 2d,e, *p* < 0.01, *p* < 0.001). Consistently, RT‐qPCR revealed significantly upregulated mRNA levels of these stemness markers in 3D‐cultured LNCs (Figure 2f, *p* < 0.01). To assess cellular aging, SA‐β‐gal staining was performed. Dissociated cells from both culture conditions were replated into six‐well plates and cultured until cells fully spread (approximately 24 h) prior to staining. Markedly more blue‐staining cells were observed in 2D‐cultured cells compared with 3D‐cultured cells (Figure 2g,h, *p* < 0.001). Furthermore, expression of senescence‐associated markers p16, p21, p53, and the DNA damage marker γ‐H2AX was evaluated by IF, which demonstrated significantly decreased expression of these markers in 3D‐cultured LNCs (Figure 2i,j, *p* < 0.05, *p* < 0.01). These findings indicated that 3D culture effectively enhanced proliferative capacity, maintained stemness characteristics, and suppressed cellular aging, representing a superior culture environment for preserving LNC functional properties.

### 3.3 3D Culture Attenuates LNC Aging Through Subpopulation Remodeling

**FIGURE 3:**
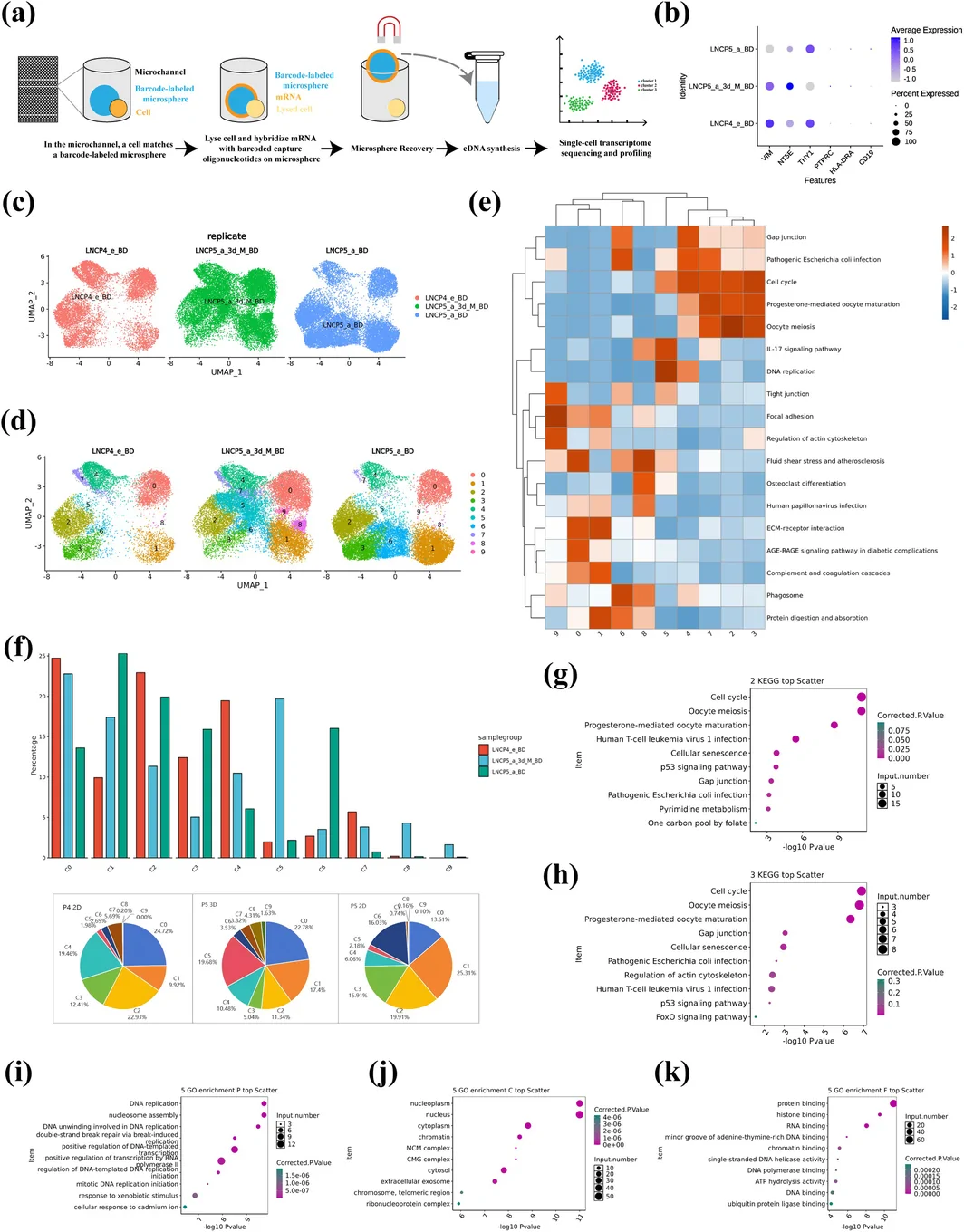
3D culture attenuates LNC aging through subpopulation remodeling. (a) The scRNA‐seq workflow for 2D P4, 2D P5, and 3D P5 LNCs. (b) Dot plot of LNC marker expression, showing positive markers (Vim, CD73, CD90) and negative markers (CD45, HLA‐DR, CD19). The dot size represents the percentage of cells expressing the gene, and the color intensity is average expression levels. (c) UMAP visualization of all cells colored by sample origin (left) and replicate identity (middle, right). (d) UMAP visualization identifying 10 clusters (C0–C9). (e) Heatmap displaying the top enriched GO biological process termed across the 10 clusters, with color intensity indicating enrichment significance (*z*‐score). (f) Bar charts (top) and pie charts (bottom) showing the relative proportions of each cluster across 2D P4, 2D P5, and 3D P5. (g, h) KEGG pathway enrichment scatter plots for C2 (g) and C3 (h). (i–k) GO enrichment scatter plots for C5 showing significant associations with DNA replication (i), nuclear division (j), and cell cycle processes (k). The dot size represents gene count, and color intensity represents the adjusted *p*‐value.

To investigate the mechanism underlying the reversal of aging in 3D culture, we performed scRNA‐seq on samples of 2D P4 LNCs, 2D P5 LNCs, and 3D P5 LNCs (Figure 3a). These cells all expressed positive markers such as Vim, CD73, and CD90, but did not express CD45, HLA‐DR, or CD19 (Figure 3b and Figure S1). Unsupervised clustering analysis via UMAP identified 10 distinct transcriptional clusters (C0–C9) across all samples (Figure 3c,d). GO functional enrichment analysis revealed significant functional differences among these clusters. C0, C1, C6, C8, and C9 showed high enrichment in cell adhesion and ECM signaling pathways, including gap junctions, ECM‐receptor interactions, and focal adhesions. This indicated their involvement in cell‐matrix signaling. C2, C3, C4, and C7 exhibited significant expression of cell cycle‐related genes (including H2AFZ, CKS1B, and CCNB1) and were in a proliferative state. Concurrently, C2 and C3 also displayed enrichment in cellular senescence and p53 signaling pathways, indicating that these two clusters were in a transitional state between active proliferation and stress‐induced senescence. C5 was uniquely characterized by enrichment in DNA replication processes (Figure 3e and Figure S2). We calculated the proportions of each sample cluster and observed significant changes in cluster distribution under different culture conditions (Figure 3f). C2 and C3, which were highly abundant in 2D‐cultured LNCs, were markedly reduced in 3D‐cultured LNCs, whereas C5 was significantly increased under the 3D environment. KEGG analysis confirmed that C2 and C3 exhibited senescence‐like characteristics (Figure 3g,h). GO enrichment analysis of C5, which was significantly expanded in 3D culture, revealed strong associations with DNA replication, cell cycle progression, and mitotic nuclear division (Figure 3i–k). This was consistent with a highly proliferative, rejuvenated cellular state. These analyses indicated that 3D culture attenuated cellular aging by reducing senescence‐prone clusters (C2 and C3) while expanding the proliferative and actively DNA‐replicating cluster (C5).

### 3.4 FOSL1 Plays a Pivotal Role in Regulating Cellular Aging Within 3D Culture

**FIGURE 4:**
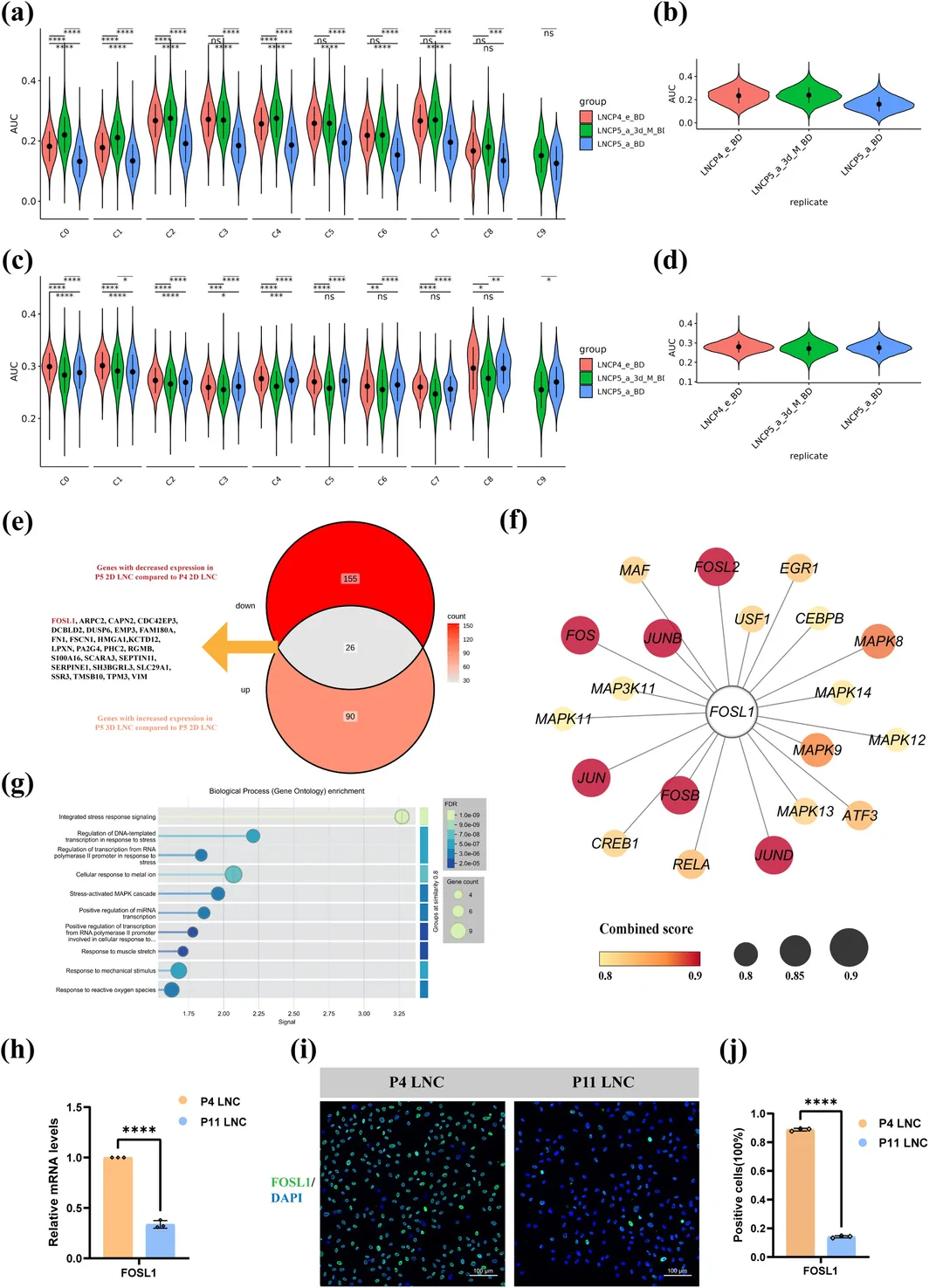
Identification of FOSL1 as a pivotal regulator mediating the anti‐senescence effects of 3D culture. (a, b) Gene set scoring analysis showing pluripotency‐associated transcriptional signatures across distinct clusters (a) and overall area under the curve (AUC) scores (b) in 2D P4, 2D P5, and 3D P5 LNCs. Gene signatures were derived from Gao et al. (2023). (c, d) Senescence‐associated gene expression signatures across clusters (c) and overall AUC scores (d), demonstrating significant suppression of aging signatures in 3D‐cultured LNCs compared to 2D counterparts. (e) Venn diagram illustrating the intersection of genes downregulated in 2D P5 versus 2D P4 LNCs (passage‐induced aging) and genes upregulated in 3D P5 versus 2D P5 LNCs (3D‐mediated rejuvenation), revealing 21 candidate regulators, including FOSL1. (f) Protein–protein interaction network showing FOSL1‐associated transcription factors, including AP‐1 complex members (FOS and JUN) and stress‐responsive regulators (MAF and EGR1). (g) GO biological process enrichment analysis of FOSL1‐correlated genes, showing significant enrichment in protein folding, stress response, and cellular homeostasis pathways. (h) Relative *FOSL1* mRNA levels in early‐passage (P4) and late‐passage (P11) LNCs determined by RT‐qPCR. (*n* = 3) (i) IF staining of FOSL1 (green) and DAPI (blue) staining in P4 and P11 LNCs. Scale bars, 100 μm. (*n* = 3) (j) Quantification of the percentage of FOSL1‐positive cells. (*n* = 3) Data are presented as mean ± SD from three independent experiments. Statistical significance was determined by Student's *t*‐test: ****p < 0.0001; ***p < 0.001; **p < 0.01; *p < 0.05; ns, not significant.

We utilized the transcriptomic profiles established by Gao et al. (2023) (Table S4). Through gene set scoring analysis, we investigated the regulatory mechanisms underlying the aging differences between 2D‐ and 3D‐cultured LNCs. The scoring results showed that, compared with 2D P4 and 2D P5 LNCs, the pluripotency‐related transcriptomic profiles of 3D P5 LNCs were significantly upregulated (Figure 4a,b). The expression of aging‐related genes was significantly reduced in 3D P5 LNCs (Figure 4c,d). These results indicated that 3D culture effectively attenuated age‐related transcriptional changes. To identify key regulatory factors mediating the rejuvenating effects of 3D culture, we performed differential expression analysis of the whole transcriptome. We employed a dual‐criterion screening strategy, identifying genes downregulated in 2D P5 LNCs relative to 2D P4 LNCs and genes upregulated in 3D P5 LNCs relative to 2D P5 LNCs. Subsequently, we identified the intersection of these two sets of genes. This approach revealed 21 candidate rejuvenation‐associated regulators, including FOSL1 (FOS‐like antigen 1) (Figure 4e). To demonstrate the single‐cell relationship between FOSL1 and the C5 cluster, we performed GO enrichment analysis of genes upregulated in the C5 cluster under 3D versus 2D culture conditions, which revealed significant enrichment of the transcription factor AP‐1 complex (Figure S3); since FOSL1 is a core subunit of AP‐1, this functionally links FOSL1 upregulation to C5 cluster identity. The overall GO enrichment across all clusters is provided for comparative context (Figure S4). Furthermore, we compiled an intersection table of genes upregulated in 3D culture (all clusters) and downregulated in senescent P5 versus P4 cells (Table S5). While FOSL1 has been previously implicated in promoting cell proliferation (Wu et al. 2021), its specific role in anti‐senescence regulation remains to be elucidated. To investigate whether FOSL1 regulates anti‐senescence gene expression, protein interaction network analysis was performed. This revealed that FOSL1 interacts with multiple transcription factors, including AP‐1 complex members (FOS and JUN) and stress‐responsive regulators (MAF and EGR1) (Figure 4f,g). This indicated its role as a regulatory hub in cellular stress adaptation and proteostasis. To validate the expression dynamics of FOSL1 during cellular aging, we examined its expression levels in early‐passage (P4) and late‐passage (P11) LNCs. Both FOSL1 mRNA levels and protein expression were significantly reduced in P11 compared with P4 LNCs (Figure 4h–j, *p* < 0.0001). These findings collectively provided evidence that FOSL1 had the potential to act as a critical regulator of cellular aging.

### 3.5 Functional Validation of FOSL1 as an Anti‐Senescence Regulator in LNCs

**FIGURE 5:**
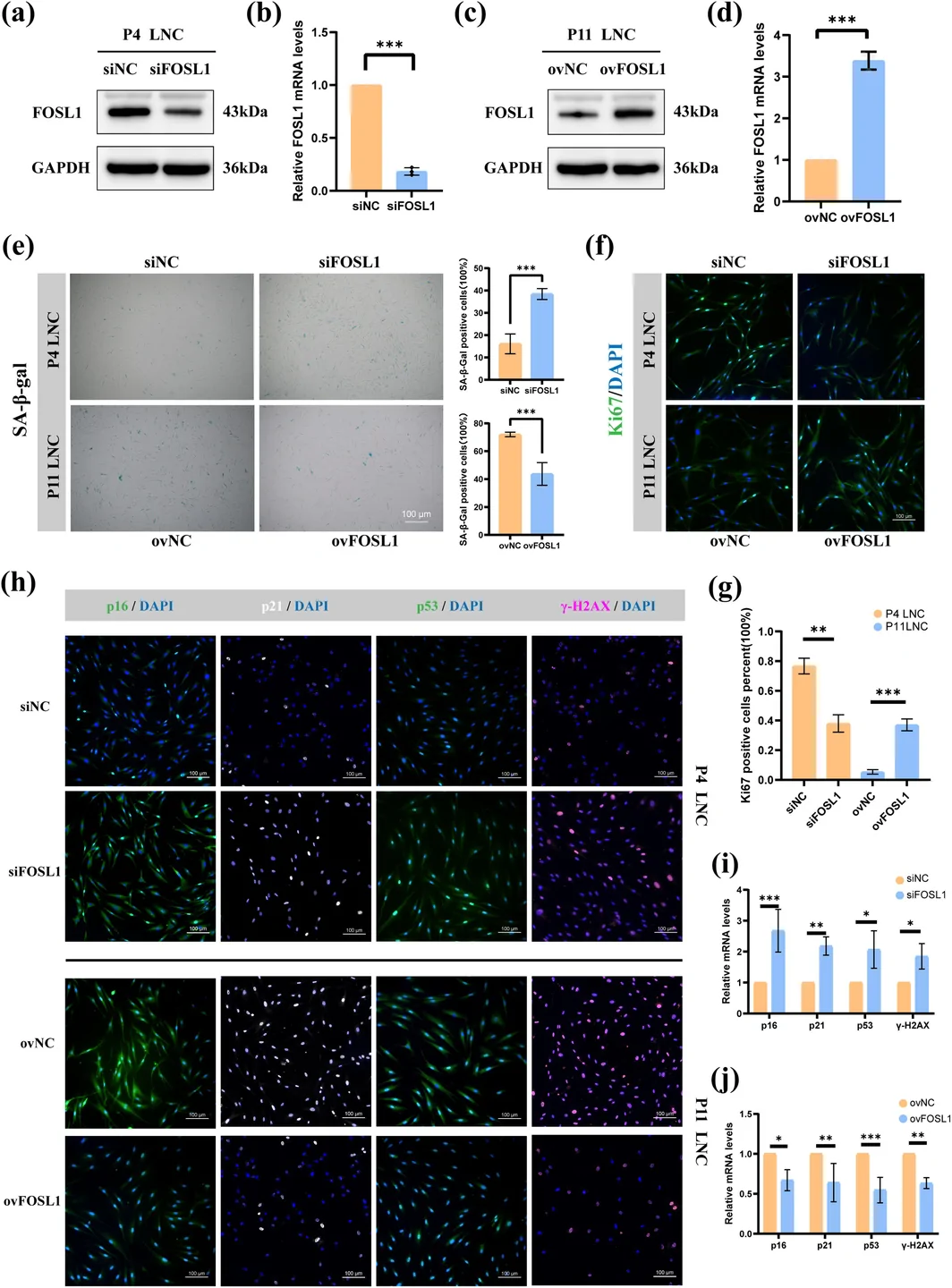
Functional validation of FOSL1 as an anti‐senescence regulator in LNCs. (a) WB of FOSL1 protein expression in P4 LNCs transfected with FOSL1‐specific siRNA (siFOSL1) or negative control (siNC) (*n* = 3). (b) RT‐qPCR of FOSL1 mRNA levels in P4 LNCs after siRNA transfection (*n* = 3). (c) WB of FOSL1 protein expression in P11 LNCs transfected with FOSL1 overexpression plasmid (ovFOSL1) or vector control (ovNC) (*n* = 3). (d) Relative FOSL1 mRNA levels in P11 LNCs after overexpression (*n* = 3). (e) Representative images and quantification of SA‐β‐gal‐positive LNCs. Scale bars, 100 μm (*n* = 3). (f) IF staining of Ki67 (green) and DAPI (blue) staining in P4 and P11 LNCs. Scale bars, 100 μm (*n* = 3). (g) Quantification of Ki67‐positive cell percentage (*n* = 3). (h) IF images of senescence‐associated markers p16, p21, p53, and γ‐H2AX (all green) with DAPI (blue) in P4 and P11 LNCs. Scale bars, 100 μm (*n* = 3). (i, j) Relative mRNA expression levels of p16, p21, p53, and γ‐H2AX in P4 (i) and P11 (j) LNCs determined by RT‐qPCR (*n* = 3). Data are presented as mean ± SD from three independent experiments. Student's *t*‐test determined statistical significance: ***p < 0.001; **p < 0.01; *p < 0.05.

To validate the role of FOSL1 in regulating cellular aging, we performed knockdown and overexpression experiments in early‐passage (P4) and late‐passage (P11) LNCs, respectively. Transfection with FOSL1‐specific siRNA (siFOSL1) in P4 LNCs significantly reduced FOSL1 protein and mRNA levels compared to the negative control (siNC) (Figure 5a,b, *p* < 0.001; Figure S5), whereas overexpression of FOSL1 (ovFOSL1) in P11 LNCs markedly elevated FOSL1 expression compared to the vector control (ovNC) (Figure 5c,d, *p* < 0.001; Figure S5). Subsequently, we assessed the impact of FOSL1 regulation on cellular aging through SA‐β‐gal staining. Knockdown of FOSL1 in P4 LNCs significantly increased the percentage of SA‐β‐gal‐positive cells compared to controls. Conversely, overexpression of FOSL1 in P11 LNCs substantially reduced the proportion of senescent cells (Figure 5e, *p* < 0.001). FOSL1 knockdown markedly decreased Ki67‐positive cell percentages in P4 LNCs, whereas FOSL1 overexpression restored proliferative capacity in P11 LNCs (Figure 5f,g, *p* < 0.01, *p* < 0.001). Furthermore, we assessed the expression of established senescence‐associated molecular markers. IF staining revealed that FOSL1 knockdown in P4 LNCs upregulated the expression of p16, p21, p53, and γ‐H2AX, while FOSL1 overexpression in P11 LNCs downregulated these markers (Figure 5h). Quantitative RT‐PCR analysis confirmed that siFOSL1 significantly elevated mRNA levels of p16, p21, p53, and γ‐H2AX in P4 LNCs (Figure 5i, *p* < 0.05), whereas ovFOSL1 significantly suppressed the expression of these genes in P11 LNCs (Figure 5j, *p* < 0.05). Collectively, these data demonstrated that FOSL1 functioned as a negative regulator of cellular aging, with its depletion accelerating aging phenotypes in young cells and its restoration rejuvenating aging cells.

### 3.6 FOSL1 Affects Mitochondrial Function

**FIGURE 6:**
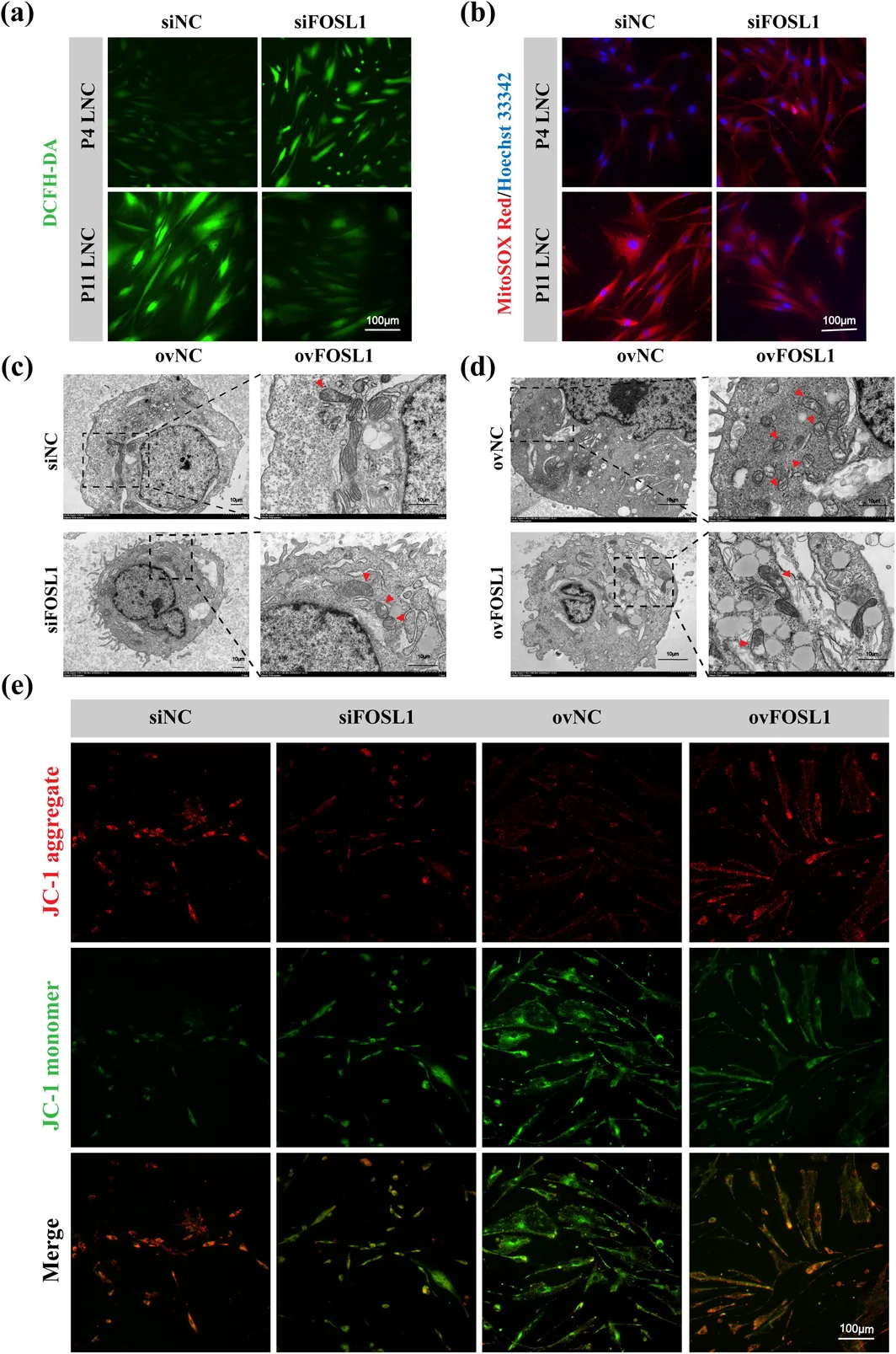
FOSL1 affects mitochondrial function. (a) IF images of intracellular ROS detection by DCFH‐DA (green) staining in P4 and P11 LNCs. Scale bars, 100 μm (*n* = 3). (b) IF images of mitochondrial superoxide detection by MitoSOX Red staining (red) with Hoechst 33342 (blue) nuclear counterstaining in P4 and P11 LNCs. Scale bars, 100 μm (*n* = 3). (c, d) TEM images of mitochondrial ultrastructure in P4 LNCs (c) and P11 LNCs (d). Red arrows indicate swollen mitochondria with disrupted cristae or vacuolization. Scale bars, 2 μm (low magnification) and 500 nm (high magnification) (*n* = 3). (e) IF images of JC‐1 staining, showing red fluorescent aggregates (polarized mitochondria) and green monomers (depolarized mitochondria) with merged images. Scale bars, 100 μm. (*n* = 3), biological replicates per group.

To investigate whether FOSL1‐mediated anti‐senescence effects involve mitochondrial homeostasis, we examined ROS levels, mitochondrial morphology, and mitochondrial membrane potential following FOSL1 modulation. DCFH‐DA staining results showed that FOSL1 knockdown in P4 LNCs significantly increased intracellular ROS levels. Conversely, FOSL1 overexpression in P11 LNCs significantly reduced ROS accumulation (Figure 6a). We subsequently performed MitoSOX Red staining to detect mitochondrial superoxide levels. FOSL1 knockdown increased mitochondrial superoxide anion production in P4 LNCs. Restoration of FOSL1 expression in P11 LNCs reduced mitochondrial superoxide levels (Figure 6b). We further evaluated mitochondrial ultrastructure using transmission electron microscopy (TEM). In P4 LNCs, FOSL1 silencing led to mitochondrial swelling, disruption of cristae structure, and vacuolization (as indicated by red arrows) (Figure 6c). Overexpression of FOSL1 in P11 LNCs improved mitochondrial damage, restored ordered cristae structure, and alleviated mitochondrial swelling (Figure 6d). We also assessed mitochondrial membrane potential using JC‐1 staining. FOSL1 knockdown in P4 LNCs caused fluorescent aggregates to shift from red to green monomers. In contrast, FOSL1 overexpression in P11 LNCs partially restored mitochondrial membrane potential (Figure 6e). Collectively, these findings demonstrated that FOSL1 was essential for maintaining mitochondrial function, with its depletion accelerating mitochondrial dysfunction and its overexpression rescuing aging‐associated mitochondrial impairment.

## 4 Discussion

As a key component of the limbal microenvironment, LNCs participate in maintaining corneal epithelial homeostasis and promoting wound healing (González and Deng 2013; Shen et al. 2025). However, replicative aging during the in vitro expansion process limits their clinical application and therapeutic efficacy. In this study, we demonstrated that a 3D Matrigel‐based culture system effectively reversed the replicative aging of LNCs, consistent with previous observations in MSCs (Cheng et al. 2012; Guo et al. 2014). This rejuvenation was manifested as restored stemness marker expression (SOX2, OCT4, and NANOG), heightened proliferative activity (Ki67), and attenuated aging signatures (reduced SA‐β‐gal activity and downregulated p16, p21, p53, γ‐H2AX). Through scRNA‐seq profiling and functional analysis, we identified FOSL1 as a pivotal regulator mediating these rejuvenation effects. Functional validation showed that FOSL1 overexpression in aged cells reduced ROS levels, preserved mitochondrial membrane potential, and maintained mitochondrial homeostasis. Consequently, FOSL1 overexpression alleviated replicative aging and mitochondrial dysfunction. Conversely, FOSL1 knockdown exerted the opposite effects. These findings established FOSL1 as a novel therapeutic target for enhancing the functionality of aged LNCs and provided a robust mechanistic basis for optimizing culture conditions in regenerative medicine and cell‐based therapies.

Although 2D culture systems are widely used, they fail to fully replicate the complex microenvironment found in vivo, which may lead to premature senescence and loss of stem cell properties during long‐term expansion (Ho et al. 2019; Bara et al. 2014). Our results demonstrated that 3D culture promoted the spontaneous self‐organization of LNCs into spheroids, creating a microenvironment that closely mimicked the native limbal niche. scRNA‐seq analysis revealed distinct functional heterogeneity among LNC subpopulations, with 3D culture selectively expanding a proliferative, DNA replication‐active subpopulation (C5) while concomitantly reducing the proportion of senescence‐prone subpopulations (C2 and C3) characterized by cellular senescence and p53 signaling activation. This observation aligned with previous reports indicating that 3D culture preserved the “youthful” state of MSCs by enhancing cell–cell and cell‐ECM interactions (Li, Tang, et al. 2025; Krasnova et al. 2023), which activated survival signaling pathways including PI3K/AKT and MAPK cascades while suppressing stress‐induced senescence programs (Lee et al. 2020; Xu et al. 2018; Liu et al. 2011; Chan et al. 2021).

To elucidate the molecular mechanisms underlying 3D‐mediated rejuvenation, we conducted differential expression analysis and identified FOSL1 as a candidate regulator. While FOSL1 has been previously implicated in promoting cell self‐renewal and proliferation, its specific role in antagonizing cellular aging remains unexplored (Wu et al. 2021; Zeng et al. 2025; Al‐Khayyat et al. 2023). As a pivotal member of the AP‐1 transcription factor family (Shaulian and Karin 2002; Belguise et al. 2005; Sobolev et al. 2022), FOSL1 may orchestrate a dual regulatory mechanism in cellular responses to oxidative stress: it enhances ROS scavenging by activating the NRF2 antioxidant pathway and upregulating key antioxidant enzymes such as glutathione peroxidase 4 (GPX4), while concurrently suppressing ROS generation by preserving mitochondrial health (Shao et al. 2023; Ursini and Maiorino 2020; Wang, Gong, et al. 2024; Qin et al. 2024; Guo et al. 2025). Our study revealed that FOSL1 expression progressively declined with cellular aging. Protein interaction network analysis further indicated that FOSL1 functioned as a regulatory hub, interacting with AP‐1 complex members (FOS, JUN) and stress‐responsive transcription factors (MAF, EGR1). These interactions suggested that FOSL1 may coordinate cellular stress adaptation and proteostasis, thereby maintaining the functional integrity of the limbal microenvironment. Functional validation experiments provided robust evidence for the antiaging effects of FOSL1. Knocking down FOSL1 in early‐passage LNCs accelerated the onset of aging phenotypes, while overexpressing FOSL1 in aged LNCs effectively reversed aging markers, restored proliferative potential, and maintained mitochondrial membrane potential. These bidirectional regulatory experiments established a relationship between FOSL1 expression and the cellular aging state, thereby positioning FOSL1 as a key negative regulator of aging.

Mitochondrial dysfunction represents a hallmark of cellular aging (Passos et al. 2007; Sharma et al. 2019; Houtkooper et al. 2013). Our study revealed that the antiaging effects of FOSL1 were associated with the maintenance of mitochondrial homeostasis. FOSL1 knockdown led to elevated intracellular and mitochondrial ROS levels, mitochondrial swelling accompanied by cristae disruption, and reduced mitochondrial membrane potential. Overexpression of FOSL1 in aged cells improved mitochondrial structure and function. These findings suggest that FOSL1 counteracts oxidative stress‐induced mitochondrial dysfunction, thereby breaking the vicious cycle between ROS accumulation and cellular damage.

Our findings have implications for the clinical application of LNCs. Currently, the use of LNCs for therapeutic purposes is limited by the insufficient cell proliferation capacity of traditional 2D culture, which fails to yield adequate cell numbers. Our study suggested that 3D culture helped maintain the stem cell properties of LNCs and delayed their aging, thereby providing a scalable method for preparing cells for the treatment of LSCD and corneal surface diseases. By rejuvenating aged LNCs and selectively expanding a proliferative subpopulation, 3D culture could provide an abundant, standardized cellular product for bioengineered corneal constructs or direct subconjunctival injection. Furthermore, activating FOSL1 is a viable approach. Whether through genetic or pharmacological means, it is possible to revitalize senescent LNCs.

Numerous studies have confirmed that FOSL1 acts as an oncogenic driver in various epithelial tumors, promoting epithelial‐mesenchymal transition, invasion, and treatment resistance (Khedri et al. 2024; Zhu et al. 2019; Zhang et al. 2021). However, existing evidence suggests that FOSL1 is not only a tumor‐associated protein but also a transcription factor physiologically expressed in LESCs. Multi‐omics analyses have identified FOSL1 as an LESC‐specific transcription factor that maintains the identity of corneal epithelial cells (Smits et al. 2023). Notably, FOSL1 expression is significantly downregulated in keratoconus, a degenerative corneal disease characterized by loss of epithelial integrity and impaired oxidative stress responses (Shinde et al. 2020). In this pathological context, FOSL1 functions as a transcriptional regulator of the NRF2 antioxidant pathway, consistent with our observation that FOSL1 overexpression in aged LNCs restores mitochondrial homeostasis and reduces ROS levels. Therefore, in the limbal microenvironment, FOSL1 appears to exert a homeostatic regulatory function rather than an oncogenic one. Further research is needed to evaluate whether FOSL1 activation can be naturally confined to the anatomical site of the lesion through local cell therapy rather than systemic pharmacological activation.

However, this study has several limitations. Although our cellular experiments confirmed the anti‐aging effects of FOSL1, these findings still need to be validated in animal models. The specific downstream targets of FOSL1 and the precise molecular pathways linking FOSL1 to mitochondrial protection remain to be fully elucidated. In future studies, we will focus on establishing animal models of corneal injury or LSCD to evaluate the therapeutic effects of LNCs that upregulate FOSL1. Moreover, we will conduct in‐depth investigations into the upstream and downstream pathways of FOSL1. This will support the clinical application of LNCs in the treatment of corneal disorders.

## 5 Conclusion

This study demonstrated that a Matrigel‐based 3D culture system more closely mimicked the native limbus than traditional 2D culture. The 3D environment promoted spontaneous spheroid formation in LNCs and effectively reversed their replicative aging. The results showed that 3D‐cultured LNCs exhibited significantly increased FOSL1 expression. FOSL1 is a transcription factor of the AP‐1 family and a key negative regulator of cellular aging. Functional validation indicated that knocking down FOSL1 accelerated senescence in early‐passage cells, whereas overexpression of FOSL1 in senescent LNCs alleviated senescence‐associated phenotypes and enhanced proliferative capacity. These findings establish FOSL1 as a potential novel therapeutic target for enhancing LNC function and provide a basis for an LNC‐based therapeutic strategy for LSCD.

## Author Contributions

X.W. and S.L. conceived and designed the study, performed experiments, analyzed data, and wrote the manuscript. Z.L., X.G., J.S., T.Z., S.L., X.H., W.W., and L.X. performed experiments and analyzed data. G.L. and X.Z. supervised the project and revised the manuscript.

## Funding

This research was funded and supported by grants from the National Natural Science Foundation of China (No. 82471050, 82070936), the Science and Technology Talent Service Enterprise Project of Department of Science and Technology of Hubei Province (No. 2024DJC066), and the Research Project of Hubei Provincial Health Commission (No. WJ2021ZH0005).

## Conflicts of Interest

The authors declare no conflicts of interest.

## Supporting information


**Table S1:** Donor information.
**Table S2:** Antibodies for immunofluorescence staining and western blotting.
**Table S3:** Forward and reverse primers used for qPCR gene expression analysis.
**Figure S1:** LNC marker expression in UMAP feature plots. UMAP feature plots showing high expression of positive LNC markers (VIM, NT5E, THY1) and absent expression of negative immune markers (PTPRC, HLA‐DRA, CD19) across the sequenced cell population.
**Figure S2:** Top 3 marker genes distinguishing each transcriptional cluster. Dot plot showing the highest differentially expressed genes for clusters C0–C9 identified by Seurat FindAllMarkers analysis. For each cluster, the top 3 genes with the highest average log2 fold change (avg_log2FC) and adjusted *p* < 0.05 are displayed. Dot size represents the percentage of cells expressing each gene, and color intensity indicates average expression level.
**Table S4:** Lists of genes used to the gene set scoring analysis.
**Figure S3:** GO enrichment analysis of upregulated genes in Cluster 5 (C5) under 3D versus 2D culture conditions. Scatter plot showing the top significantly enriched GO cellular component terms for genes upregulated in the C5 subpopulation following 3D Matrigel culture compared to 2D culture. Bubble size represents the number of enriched genes (Input number), and color intensity indicates the corrected *p* value (−log_10_ scale).
**Figure S4:** GO enrichment analysis of upregulated genes in all clusters under 3D versus 2D culture conditions. Scatter plot showing the top significantly enriched GO cellular component terms for genes upregulated in the all clusters following 3D Matrigel culture compared to 2D culture. Bubble size represents the number of enriched genes (Input number), and color intensity indicates the corrected *p* value (−log_10_ scale).
**Table S5:** The intersection of genes downregulated in 2D P5 versus 2D P4 LNCs and genes upregulated in 3D P5 versus 2D P5 LNCs.
**Figure S5:** WB of FOSL1 protein expression. Validation of FOSL1 knockdown and overexpression efficiency in LNCs.

## Data Availability

The data that support the findings of this study are available from the corresponding author upon reasonable request. The scRNA‐seq data generated in this study have been deposited in the Gene Expression Omnibus (GEO) database under accession number GSE343251.
