# Supplementary material for: 3D Culture Reverses Limbal Niche Cell Replicative Aging via FOSL1 Upregulation
Source: Aging Cell. 2026 Sep 8;25(9):e70705. doi: 10.1111/acel.70705 (PMC13554720; doi:10.1111/acel.70705)
Supplement: Supplementary file 1 [file ACEL-25-e70705-s001.docx]

**Table S1. Donor information**

| **Donors** | **Age (Years)** | **Gender** | **Cause of death** |
| --- | --- | --- | --- |
| 1 | 68 | male | respiratory failure |
| 2 | 79 | male | circulatory failure |
| 3 | 67 | male | respiratory failure |
| 4 | 62 | male | intracerebral hemorrhage |
| 5 | 76 | male | respiratory failure |
| 6 | 65 | female | circulatory failure |
| 7 | 66 | female | intracerebral hemorrhage |
| 8 | 71 | male | intracerebral hemorrhage |
| 9 | 69 | male | intracerebral hemorrhage |
| 10 | 73 | male | respiratory failure |
| 11 | 76 | female | cerebral infarction |
| 12 | 64 | male | respiratory failure |
| 13 | 78 | male | circulatory failure |
| 14 | 70 | male | cerebral infarction |
| 15 | 75 | female | cerebral infarction |

**Table S2. Antibodies for immunofluorescence staining and western blotting**

| **Antibodies** | **Catalog** | **Sources** | **Dilution** |
| --- | --- | --- | --- |
| Pan-cytokeratin | ab7753 | Abcam | 1: 400 |
| Vimentin | Ab16700 | Abcam | 1: 400 |
| CD73 | 13160T | Cell Signaling | 1: 100 |
| CD90 | 66766-1-Ig | Proteintech | 1: 200 |
| Ki67 | 27309-1-AP | Proteintech | 1: 200 |
| SOX2 | 3579T | Cell Signaling | 1: 400 |
| OCT4 | 75463S | Cell Signaling | 1: 200 |
| Nanog | 4903T | Cell Signaling | 1: 200 |
| P16 | ab211542 | Abcam | 1: 200 |
| P21 | ab109520 | Abcam | 1: 500 |
| P53 | TA0879 | Abmart | 1: 200 |
| γ-H2AX | MJF3107547 | Abmart | 1: 200 |
| FOSL1 | ab252421 | Abcam | 1: 500 for IF and 1: 1000 for WB |
| GAPDH | ab181602 | Abcam | 1: 5000 for WB |
| Donkey anti-Mouse IgG (H+L) ReadyProbes^TM^ Secondary Antibody, Alexa Fluor™ 488 | R37114 | Invitrogen | 1: 400 |
| Donkey anti-Mouse IgG (H+L) ReadyProbes™ Secondary Antibody, Alexa Fluor™ 594 | R37115 | Invitrogen | 1: 400 |
| Donkey anti-Rabbit IgG (H+L) ReadyProbes™ Secondary Antibody, Alexa Fluor™ 488 | R37118 | Invitrogen | 1: 400 |
| Donkey anti-Rabbit IgG (H+L) ReadyProbes™ Secondary Antibody, Alexa Fluor™ 594 | R37119 | Invitrogen | 1: 400 |
| Goat anti Rabbit HRP | ab205718 | Abcam | 1: 2000 |
| Goat anti Mouse HRP | ab205719 | Abcam | 1: 2000 |
| Antifade Mountant with  DAPI | BL739A | Biosharp | N/A |

**Table S3. Forward and reverse primers used for qPCR gene expression analysis**

| **Gene** |  | **Primer Sequences** |
| --- | --- | --- |
| GAPDH | Forward | AGCCACATCGCTCAGACACC |
|  | Reverse | GCCCAATACGACCAAATCC |
| SOX2 | Forward | GGATAAGTACACGCTGCCCG |
|  | Reverse | TCATGCTGTAGCTGCCGTTG |
| OCT4 | Forward | TTGAGAACCGAGTGAGAGGCA |
|  | Reverse | TTGATCGTTTGCCCTTCTGGC |
| Nanog | Forward | TGAACCTCAGCTACAAACAGGTGA |
|  | Reverse | TGCCACCTCTTAGATTTCATTCTCT |
| P16 | Forward | CGCAGGTTCTTGGTCACTGT |
|  | Reverse | TGTTCACGAAAGCCAGAGCG |
| P21 | Forward | GACGAGAACGGTGGAACTTTGAC |
|  | Reverse | CAGGGCTCAGGTAGACCTTG |
| P53 | Forward | TGCGTGTGGAGTATTTGGATGAC |
|  | Reverse | CAGTGTGATGATGGTGAGGATGG |
| γ-H2AX | Forward | GAGCAGCTGGAAGATGATGA |
|  | Reverse | AGGTAGAGCTTGGCTTGTGA |
| FOSL1 | Forward | GCCTTGTGAACAGATCAGCC |
|  | Reverse | AGTTTGTCAGTCTCCGCCTG |

**Supplementary Figure 1. LNC marker expression in UMAP feature plots.** UMAP feature plots showing high expression of positive LNC markers (VIM, NT5E, THY1) and absent expression of negative immune markers (PTPRC, HLA-DRA, CD19) across the sequenced cell population.

**Supplementary Figure 2. Top 3 marker genes distinguishing each transcriptional cluster.** Dot plot showing the highest differentially expressed genes for clusters C0–C9 identified by Seurat FindAllMarkers analysis. For each cluster, the top 3 genes with the highest average log2 fold change (avg_log2FC) and adjusted *P* < 0.05 are displayed. Dot size represents the percentage of cells expressing each gene, and color intensity indicates average expression level.

**Table S4. Lists of genes used to the gene set scoring analysis**

| **Gene set** | **Genes** |
| --- | --- |
| Cellular Senescence | HSPA2; CDKN2A; SERPINE1; CDKN2B; CYP1B1; CCND1; RRAS; RHOB; FILIP1LNRG1; RAB31; CCN2; VIM; IGFBP4; MMP1; RAB5B; S100A11; GUK1; MAP2K3; MAP1LC3B; CXCL14; IRF5; CITED2; HTATIP2; CDKN2D; IGFBP3; NME2; ISG15; NDN; IGFBP2; RBL2; TSPYL5; CLTB IRF7; IGFBP7; F3; IGFBP6; TNFAIP3; TP53; IGFBP5; HBS1L; ALDH1A3; RAC1; STAT1; IFNG; IGSF3; THBS1; IFI16; ING1; CDKN1C; OPTN; RGL2; CREG1; SOD1; CRYAB; COL1A2; HPS5; RABGGTA; SMURF2; PEA15; AOPEP; RAB13; MDM2; TFAP2A; TGFB1I1; SPARC; TNFAIP2; SERPINB2; TES; CD44; IGFBP1; FN1; CDKN1A; EIF2S2; ESM1; SMPD1; GSN |
| Stem Cell Signature | NANOG; POU5F1; SOX2; DNMT1; PRMT1; EZH2; SUZ12; EED; PHF19; YAP1; HMGA1; HMGA2; FOXP1; REST; MYC; PAF1; PHF5A; SOX11; THAP11; ZEB1 |

**Supplementary Figure 3. GO enrichment analysis of upregulated genes in cluster 5 (C5) under 3D versus 2D culture conditions**

Scatter plot showing the top significantly enriched GO cellular component terms for genes upregulated in the C5 subpopulation following 3D Matrigel culture compared to 2D culture. Bubble size represents the number of enriched genes (Input number), and color intensity indicates the corrected *P* value (-log₁₀ scale).

**Supplementary Figure 4. GO enrichment analysis of upregulated genes in all clusters under 3D versus 2D culture conditions**

Scatter plot showing the top significantly enriched GO cellular component terms for genes upregulated in the all clusters following 3D Matrigel culture compared to 2D culture. Bubble size represents the number of enriched genes (Input number), and color intensity indicates the corrected *P* value (-log₁₀ scale).

**Table S5. The intersection of genes downregulated in 2D P5 versus 2D P4 LNCs and genes upregulated in 3D P5 versus 2D P5 LNCs**

| **Cluster** | **Genes** |
| --- | --- |
| Cluster 0 | ARPC2, CAPN2, CAV1, CAVIN1, CDC42EP3, CSRP1, DUSP6, EMP3, FN1, FOSL1, FSCN1, HMGA1, LPXN, PFN1, PRRX1, PTMS, RGMB, S100A16, SEMA5A, SEPTIN11, TMSB10, TPM3, VIM |
| Cluster 1 | ARPC2, CAPN2, CDC42EP3, DUSP6, EMP3, FAM20C, FOSL1, HDGF, HMGA1, KCTD12, LDHA, LGALS1, LMNA, PAMR1, PFN1, PTMS, RGMB, S100A16, SCARA3, SERPINE1, SH3BGRL3, TIMP3, TMSB10, TPM3, TRIOBP, VIM |
| Cluster 2 | AXL, CAPN2, CAV1, CDC42EP3, DNMT1, DUSP6, FAM180A, FAM20C, FOSL1, FSCN1, HMGA1, KCTD12, LDHA, LPXN, PHC2, RAB3B, RGMB, S100A16, SCARA3, SEPTIN11, SERPINE1, SLC29A1, TIMP3, TPM3, VIM |
| Cluster 3 | AXL, CAPN2, CCND1, CDC42EP3, DNMT1, DUSP6, EGFR, EMP3, FAM180A, FOSL1, FSCN1, HMGA1, HMGA2, KCTD12, LPXN, PA2G4, PHC2, PLEC, PRMT1, RGMB, RPL18, RPL35, S100A16, SACS, SCARA3, SERPINE1, SH3BGRL3, SSR3, TIMP3, TMSB10, TPM3 |
| Cluster 4 | ARPC2, AXL, CAPN2, CCDC80, CDC42EP3, DCBLD2, DUSP6, EGFR, FAM180A, FN1, FOSL1, FSCN1, GDNF, HMGA1, KCTD12, LPXN, PHC2, PRMT1, PRRX1, RGMB, S100A16, SEMA5A, SEPTIN11, SLC29A1, TPM3, VIM |
| Cluster 5 | ARL4C, DUSP6, EGFR, FAM180A, FOSL1, FSCN1, HMGA1, KCTD12, LPXN, PHC2, PRRX1, RHOB, S100A16, SCARA3, SERPINE1, SLC29A1, SSR3 |
| Cluster 6 | AXL, CAPN2, DUSP6, FAM20C, FOSL1, FSCN1, HMGA1, HSPA8, KCTD12, LPXN, PA2G4, PFN1, PHC2, RPL18, RPL35, S100A16, SERPINE1, SH3BGRL3, SSR3, TIMP3, UBE2S |
| Cluster 7 | AXL, CAPN2, CCDC80, CCND1, CDC42EP3, DCBLD2, DUSP6, FAM180A, FOSL1, FSCN1, GDNF, HMGA1, HMGA2, KCTD12, LPXN, PHC2, PNP, PPP2R3A, PRRX1, RGMB, S100A16, SEPTIN11, SLC29A1, SSR3, TIMP3, TMSB10, TPM3, VIM |
| Cluster 8 | PTMS |

**Supplementary Figure 5. WB of FOSL1 protein expression**

Validation of FOSL1 knockdown and overexpression efficiency in LNCs.
